# Expanded criteria for pretreatment staging CT in breast cancer

**DOI:** 10.1093/bjsopen/zraa006

**Published:** 2021-03-14

**Authors:** N Roszkowski, S S Lam, E Copson, R I Cutress, R Oeppen

**Affiliations:** Breast Imaging Unit, Princess Anne Hospital, University Hospital Southampton NHS Foundation Trust, Southampton, UK; Breast Imaging Unit, Princess Anne Hospital, University Hospital Southampton NHS Foundation Trust, Southampton, UK; Cancer Sciences Academic Unit, University of Southampton, Southampton General Hospital, Tremona Road, Southampton SO16 6YD, UK; Cancer Sciences Academic Unit, University of Southampton, Southampton General Hospital, Tremona Road, Southampton SO16 6YD, UK; Breast Surgery, University Hospital Southampton NHS Foundation Trust, Southampton, UK; Breast Imaging Unit, Princess Anne Hospital, University Hospital Southampton NHS Foundation Trust, Southampton, UK

## Abstract

**Background:**

There is wide variation in the approach to staging for distant metastatic disease in breast cancer. This study sought to identify factors predictive of distant metastatic disease at presentation to enable appropriate selection of patients for pretreatment CT.

**Methods:**

Data were collected retrospectively for all patients with newly diagnosed breast cancer (screening and symptomatic) over 3 years (2014–2017). Detailed demographic, pathological, biological, and management data were recorded at presentation, and outcome data were recorded after follow-up. Binomial logistic regression was used to identify variables independently associated with distant metastatic disease at presentation.

**Results:**

A total of 1377 patients with newly diagnosed breast cancer were identified, of whom 1025 had complete data; 323 staging CT examinations were performed. Distant metastases were identified at presentation in 47 (4.6 per cent). Some 30 of 47 patients with metastatic disease met established criteria for staging (T4, recurrence, symptoms of possible distant metastases), leaving 17 patients with metastatic disease potentially missed by use of these criteria alone. Multivariable analysis showed that tumour size at least 3 cm combined with sonographically abnormal axillary lymph nodes predicted a high probability of distant metastatic disease at presentation (positive predictive value 18.8 per cent, odds ratio 4.83, *P* < 0.001). Addition of this criterion increased the positive CT rate to 17.1 per cent.

**Conclusion:**

Selective pretreatment CT staging can be further optimized with the addition of tumour size at least 3 cm with abnormal axillary nodes to established staging criteria.

## Graphical Abstract

**Figure:**
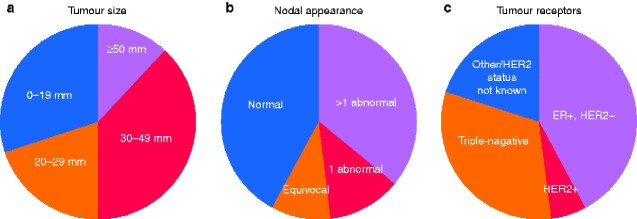

## Introduction

Identification of distant metastatic disease from breast cancer is important when planning treatment and considering patient prognosis. Selecting the correct asymptomatic patients for staging investigations can prove challenging, given the low incidence of metastases in this group at initial presentation, which is around 4 per cent^1^. Different approaches to systemic staging exist even within a single healthcare system^2^.

Current European staging guidelines are summarized in *Table 1*. These are quite disparate, with the UK National Institute for Health and Care Excellence (NICE) only clearly advocating staging of stage IV disease or patients with symptoms, whereas other groups use broader terms such as ‘aggressive biology’^3^^,^^4^^,^^7^. Many sources agree that routine staging is not recommended for asymptomatic early breast cancer^10–12^ owing to the low prevalence of metastatic disease in these patients^11^^,^^13–16^. Many of these studies, however, are based on modalities such as chest X-ray and abdominal ultrasound imaging, which are less sensitive than CT and PET–CT^17–22^. It is recognized that locally advanced disease carries a higher risk of concurrent distant metastases^22^, but this is a non-specific term, and there is no consensus regarding a threshold tumour size, level of lymph node involvement or pathological stage at which a search for distant metastatic disease should be performed.

**Table 1:** Summarized current European guidelines on systemic staging for breast cancer

	Summarized guidance on which patients should be staged
National Institute for Health and Care Excellence (NICE) CG81^3^	Assess for the presence and extent of metastases in advanced breast cancer (stage IV) using appropriate modalities
National Institute for Health and Care Excellence (NICE) QS12^4^	People with early invasive breast cancer do not undergo staging investigations for distant metastatic disease in the absence of symptoms
The Royal College of Radiologists (RCR)^20^ (Guidelines adopted by The Association of Breast Surgery (ABS)^19^)	Indications for staging: • T3 and T4 primary cancers • > 4 abnormal nodes at axillary ultrasonography or > 4 macrometastatic nodes at axillary surgery • If symptoms raise the suspicion of metastatic disease At present, there is no evidence base for carrying out staging before neoadjuvant chemotherapy in <T2 tumours with <N1 disease
London Cancer Surgical guidelines^21^	Indications for staging using CT and isotope bone scan: • Symptoms suggestive of metastases • Recurrent disease • Significant nodal involvement (e.g., ≥ 4 nodes) • As part of an approved clinical trial • Inflammatory breast cancer • Locally advanced disease • Large primary tumours (e.g., > 5cm)
European Society for Medical Oncology (ESMO)^5^	Staging can be considered for patients with: • Clinically positive axillary nodes • Large tumours (e.g., ≥ 5 cm) • Aggressive biology • Clinical signs, symptoms or laboratory values suggesting presence of metastases
National Comprehensive Cancer Network (NCCN)^32^	Consider staging with body CT, bone scan and (optional) PET–CT for: • Signs or symptoms of possible metastases • Stage IV disease • Inflammatory breast cancer • Work-up before preoperative systemic therapy • >4 positive axillary nodes at surgery Routine systemic staging is not indicated for early breast cancer in the absence of symptoms

NICE, National Institute for Health and Care Excellence; CG, Clinical Guideline; QS, Quality Standard; ESMO, European Society for Medical Oncology; ABS, Association of Breast Surgery; NCCN, National Comprehensive Cancer Network.

Although symptoms of possible metastatic disease, recurrent and T4 disease seem widely advocated^5^^,^^6^^,^^8^, some groups now include T3 disease (tumour size 5 cm or more)^5–7^ and some include unexpected heavy burden of nodal involvement (such as at least 4 nodes) at axillary surgery as justification for investigation for distant metastases^5^^,^^6^.

CT of the chest, abdomen and pelvis is widely used^5^, yet recent studies in asymptomatic women found a higher false-positive rate (10–25 per cent in 2 studies) than true-positive rate (2–9 per cent) for distant metastases when used in only early-stage disease, and concluded that a more selective approach was necessary^23–25^. PET–CT appears to increase the rate of detection of distant disease, but is currently recommended only where other investigations are suspicious but not diagnostic for metastases^3^^,^^20^^,^^22^.

The relevance of locoregional disease in predicting the likelihood of distant disease has not been studied extensively. A recent analysis of 204 patients with stage I or II breast cancer who underwent staging with CT and/or MRI and/or bone scan identified lymph node involvement and stage IIb disease as risk factors for an increased incidence of distant metastases^19^. An abstract^26^ published in 2019 reported a 10 per cent incidence of distant disease among 163 patients selected for neoadjuvant chemotherapy who underwent staging CT, as well as a correlation between increasing T category and metastatic disease in patients with clinically node-negative disease. Two other studies^27^^,^^28^ also found similar significant correlations between the presence of metastatic disease and increasing tumour size and nodal involvement. These studies have a variety of limitations including sample size, heterogeneity of imaging used and selection biases, for example including only those selected for neoadjuvant chemotherapy.

The present study aimed to identify factors predictive of distant metastatic disease at presentation in order to refine current guidelines and select appropriate patients for CT staging.

## Methods

Data were collected for all patients presenting with new invasive breast cancer (including first diagnoses and recurrent disease) at a UK teaching hospital over a 3-year period, from January 2014 to January 2017. The patients studied were a mixed symptomatic and National Health Service (NHS) Breast Screening Programme cohort. The study was registered electronically and approved on the Trust Safeguard system as Service Evaluation (SEV0140) in accordance with local governance policy. No other ethical permissions were required because the study evaluated the current service using existing data, an intervention already in use according to established local protocols, and involved no allocation and no randomization.

Information was extracted retrospectively from computer-based records of radiology and pathology reports, clinical letters and the radiology information system. A panel of demographic, radiological, pathological, and biological parameters, along with staging investigations, surgical and systemic management, and outcomes were recorded at presentation and during follow-up. For follow-up, records were examined from the time of diagnosis up to the time of data collection. The TNM version from the eighth edition of the AJCC cancer staging manual was used^29^. A standardized data collection pro forma was used (*Table S1*). Data were collated and analysed in an Excel^**^®^**^ spreadsheet (Microsoft, Redmond, Washington USA).

Standard practice was for all patients with breast cancer to be investigated with mammography, breast ultrasound imaging and axillary ultrasonography in accordance with NICE guidance^4^^,^^30^. Axillary lymph nodes were categorized as abnormal if they demonstrated any of the following features: entirely hypoechoic (loss of fatty hilum), cortex larger than 3 mm, focal cortical bulge, short–long axis ratio over 0.5 (rounded), or entirely replaced by a mass. If the operator was unsure, nodes could be classified as equivocal at their discretion. In all instances of abnormal or equivocal nodes, standard practice was to perform needle sampling of the nodes where practicable.

Results of staging CT examinations were stratified as: no distant metastases, definite evidence of distant metastases, indeterminate findings later proven not to be metastatic, or indeterminate findings later proven to be distant metastases. Patients with indeterminate features that were subsequently shown not to be metastatic were considered to have false-positive/incidental findings. Scans were classified as either pretreatment or postoperative, and were only recorded as such if done within 3 months of the diagnosis or surgery respectively. Investigations performed later in the patient pathway were not classified as initial staging examinations.

Patients with non-invasive disease or with incomplete data because of treatment provision elsewhere were excluded. Before binomial logistic regression analyses, further exclusions were applied to produce a reliable data set: these were patients with no imaging, occult tumour, tumour size not recorded, no axillary ultrasonography, no breast core biopsy, lost to follow-up abroad, and another concurrent metastatic cancer during the study period (*Fig. S1*). Those with a history of previous ipsilateral breast cancer were excluded from all regression analyses, except analyses looking specifically at possible ipsilateral recurrence.

### Statistical analysis

Positive (PPV) and negative predictive values were derived systematically for each variable. Binomial logistic regression analyses were performed using RegressIt™ software (Robert Nau, Professor Emeritus in the Fuqua School of Business at Duke University, North Carolina, USA) to identify variables independently associated with metastatic disease at presentation. Odds ratios (ORs), 95 per cent confidence intervals, *P* values, and area under the receiver operating characteristic (ROC) curve were calculated. Results were considered statistically significant at *P* < 0.050 (2-sided). Based on the results of multivariable analysis, data modelling was undertaken to calculate the projected number of CT examinations and positive pick-up rate of these for different sets of staging criteria.

## Results

Over a 3-year period, 1377 patients with a new diagnosis of invasive breast cancer were seen. There were 352 with incomplete data who were excluded. Of the remaining 1025 patients, the pathways of presentation were: 394 patients via the NHS Breast Screening Programme, 604 via symptomatic one-stop clinics, nine via surveillance imaging for either high risk family history or post-cancer surveillance, and 18 patients were incidentally found to have a breast mass on cross-sectional imaging. There were five men and 1020 women. The mean age at presentation was 62.6 (range 26–99) years. Other patient features and disease characteristics are shown in *Table 2*.

**Table 2:** Patient features and disease characteristics at presentation of 1025 patients in data set

	No. of patients	% staged with CT before treatment or after surgery
	(*n* = 1025)	
**Personal or family history**		
Previous ipsilateral breast cancer	51 (5.0)	77
Previous contralateral breast cancer	38 (3.7)	55
Low- or moderate-risk family history	119 (11.6)	29.4
High-risk family history (including *BRCA*)	12 (1.2)	75
**T category**		
T1	482 (47.0)	19.1
T2	389 (38.0)	36.5
T3	45 (4.4)	51
T4	86 (8.4)	65
**Specific imaging characteristics**		
Inflammatory cancer	13 (1.3)	100
Multifocal cancer	185 (18.0)	50.3
Abnormal or equivocal axillary nodes on ultrasonography	267 (26.0)	73.0
**Histology**		
Invasive ductal carcinoma of no special type	777 (75.8)	34.4
Invasive lobular carcinoma	87 (8.5)	25
Mixed or other disease	161 (15.7)	21.1
**Histological grade**		
1	158 (15.4)	17.7
2	536 (52.3)	28.0
3	295 (28.8)	49.2
**Specific molecular subtype**		
Triple receptor-negative	104 (10.1)	55.8
All HER2-positive disease	114 (11.1)	37.7
ER-negative, HER2-positive	48 (4.7)	52
**Other features**		
Symptoms of possible metastatic disease at presentation (e.g., bone pain)	29 (2.8)	90

Values in parentheses are percentages. HER2, human epidermal growth factor receptor 2; ER, oestrogen receptor.

Only one patient did not have imaging owing to poor performance status. In five patients, disease in the breast was occult on conventional imaging. A numerical tumour size or disease extent was given in the imaging reports for 1002 patients (97.8 per cent), allowing accurate clinical T assignment.

Some 1015 patients (99.0 per cent) underwent axillary ultrasound examination to assess nodal status. The reasons for not imaging the axilla in ten patients included high BMI, poor mobility, and poor performance status. There were 267 patients with possible or probable nodal involvement identified on axillary ultrasonography: 79 with one abnormal node; 105 with multiple (more than 1) abnormal nodes; 13 with an axillary nodal mass less than 2.5 cm; three with multiple axillary plus internal mammary, supraclavicular or contralateral axillary nodal abnormalities; and 67 with equivocal axillary nodes.

Among 267 patients with possible or probable nodal abnormality, 213 (79.8 per cent) had nodal sampling (194 fine-needle aspirations and 19 core biopsies). The reasons for not performing needle sampling in 54 patients were poor performance status with low likelihood of primary surgical management, or nodes deemed too difficult to access owing to body habitus. Needle sampling of the axillary nodes was positive for metastatic involvement in 114 (53.5 per cent), benign in 62 (29.1 per cent), and inadequate in 37 patients (17.4 per cent). For the purposes of ongoing analysis, node status was downgraded to normal where there was a benign result from patients with nodes initially categorized as equivocal or one abnormal node. Node status was upgraded or confirmed to be abnormal where metastatic involvement was detected by sampling. An insufficient sampling result was disregarded for the purposes of analysis; for these and any patient who did not undergo sampling, categorization was based on sonographic appearances only.

MRI staging was performed in selected patients in whom there was uncertainty over disease extent on conventional imaging, or a diagnosis of invasive lobular carcinoma was made before anticipated breast conservation surgery.

A total of 323 staging CT examinations were undertaken, of which 259 were pretreatment and 46 immediately postoperative. In 18, the CTs were performed for an alternative indication, prior to knowledge of a breast lesion, with a breast mass detected on the CT. Indications for staging were: T4 disease, ipsilateral recurrent cancer, symptoms of possible metastatic disease, and any number of radiologically abnormal lymph nodes, in accordance with evidence-based local guidance. Selected patients recommended for neoadjuvant chemotherapy also underwent CT staging.

There were 47 patients identified with distant metastatic disease at presentation among the 1025 studied (4.6 per cent incidence), in line with national incidence data^31^. The overall positive pick-up rate of staging CT was 14.6 per cent (47 of 323). Metastatic deposits were identified in bone (21), liver (19), lung (19), adrenal (3), distant nodes (3), retroperitoneum (2), skin (2) and omentum (1), with figures in parentheses encompassing all instances of disease at these sites from patients with single- and multiple-organ disease.

Forty-seven CT examinations (14.6 per cent) with indeterminate results (such as small lung nodules requiring follow-up or indeterminate liver lesions requiring further imaging) were later proven not to show metastatic disease. These represent incidental findings/false-positives. In total, an additional 25 CT examinations, 17 ultrasound investigations, six nuclear medicine bone scans, five PET–CT scans, five MRI examinations, three fine-needle aspirations and two plain X-rays were done to investigate these findings. There were three findings of significance (6 per cent): an ovarian mucinous cystadenoma, a chondroid lesion of uncertain malignant potential, and evidence of heart failure.

Of 46 patients undergoing CT staging after surgery (owing to unexpected pN2+ disease at axillary surgery), three had distant metastatic disease (7 per cent pick-up rate).

**Fig. 1:**
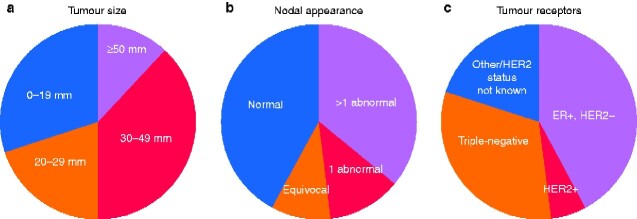
Initial presenting features of 50 tumours with delayed presentation of metastatic disease **a** Tumour size, **b** nodal appearance, **c** tumour receptors. HER2, human epidermal growth factor receptor 2; ER, oestrogen receptor.

After a mean follow-up of 26.8 months, 97 patients overall had a diagnosis of metastatic breast cancer; 50 additional patients were identified with distant disease during follow-up. Tumour characteristics of these 50 patients are shown in *Fig. 1*. Of these patients, 12 had no initial staging CT, and 38 had initial staging CT that did not identify metastatic disease. Triple-negative disease was over-represented, comprising 13 of 38 of this group with initially normal CT, whereas the prevalence of triple-negative disease in the whole data set was only 10.1 per cent.

### Predictive value of individual variables

Results of analyses to identify individual variables predictive of distant metastasis are presented in *Tables 3* and *4*. An increased incidence of distant metastatic disease at presentation was found for: inflammatory cancer; symptoms of possible metastatic disease; T4 disease; tumour size at least 3 cm, or at least 2 cm with abnormal nodes on axillary ultrasonography; oestrogen receptor (ER)-negative, human epidermal growth factor receptor 2 (HER2)-positive disease; patients staged before commencing neoadjuvant chemotherapy; and those with previous ipsilateral breast cancer. A tumour size threshold of at least 3 cm had the highest rate of detection for distant disease among tumour size criteria.

**Table 3:** Incidence of metastatic disease in relation to specific variables

	No. of patients	% initially staged	Incidence of detected distant metastases (%)	
			At presentation	After 26.8 months’ follow-up
Inflammatory breast cancer	13	100	46	62
Symptoms of possible metastases	23	100	35	65
T4 disease	75	75	21	32
Tumour size ≥ 3 cm and N1+	115	86.1	18.8	34.8
ER-negative, HER2-positive	46	54	17	26
T2+N1+	154	85.1	16.9	32.5
Before neoadjuvant chemotherapy	78	96	17	31
Ipsilateral recurrence	41	95	15	29
N1+ on axillary ultrasonography	176	80.7	13.8	31.3
Age ≤ 40 years	54	67	11	30
T3 disease	44	52	9	18
Previous contralateral cancer	36	58	8	14
Grade 3	295	49.2	8.0	19.7
Multifocal disease	172	54.1	6.4	16.9
Triple-negative	98	59	6	22
T3 N0 disease	19	16	6	16
Lobular carcinoma	85	26	6	12
Family history of breast cancer	126	34.9	0.8	8.7
Lymphovascular invasion on initial core biopsy	7	86	0	14

ER, oestrogen receptor; HER2, human epidermal growth factor receptor 2.

**Table 4:** Tumour size and nodal appearances: positive predictive values for distant metastatic disease

	No. of patients	PPV for distant metastatic disease (%)
**Tumour size (cm)(T category disregarded)**		
< 2	482	1.2
≥ 2	483	7.2
≥ 3	229	11.4
≥ 4	115	11.3
≥ 5	44	9.1
**Nodal appearance**		
Normal	748	1.9
1 abnormal node only	82	11.0
>1 (multiple) abnormal nodes	113	15.9
Axillary nodal mass > 25 mm	12	8.3

PPV, positive predictive value.

### Multivariable analyses

The results of multivariable analyses for individual variables are presented in *Table 5*. Variables showing a statistically significant independent association with the presence of metastatic disease were (in order of OR): symptomatic clinic (rather than screening) attenders (PPV 6.4 per cent, OR 19.56, *P* = 0.004, 604 patients), inflammatory breast cancer (PPV 46.2 per cent, OR 14.73, *P* < 0.001, 13 patients), symptoms of possible metastatic disease (PPV 34.8 per cent, OR 14.45, *P* < 0.001, 23 patients), T4 disease (PPV 21.3 per cent, OR 6.02–9.28, *P* <  0.001, 75 patients), N1+ disease (PPV 13.8 per cent, OR 8.58, *P* < 0.001, 176 patients), tumour size at least 3 cm (PPV 11.4 per cent, OR 4.12, *P* < 0.001, 229 patients), previous ipsilateral cancer (PPV 14.6 per cent, OR 3.69, *P* = 0.010, 41 patients), and ER-negative, HER2-positive disease (PPV 17.4 per cent, OR 3.06, *P* = 0.031, 46 patients), but not triple-negative disease (PPV 6.1 per cent, OR 1.14, *P* = 0.807, 98 patients).

**Table 5:** Logistic regression coefficient estimates for four different models describing independent relationships between five or six variables and distant metastatic disease at presentation

	Coefficient	Standard error	*z*-statistic	*P*	Lower 95% confidence interval	**Upper 95% confidence interval**	Odds ratio	VIF	Standard coefficient
**Model 1 (*n* = 988), 6 variables, AUC 0.71**									
Constant	–3.479	0.267	–13.054	< 0.001	–4.002	–2.957			
Age ≥ 60 years	–0.252	0.325	–0.777	0.437	–0.889	0.385	0.78	1.019	–0.069
High–risk family history	–18.216	6754	–0.003	0.998	–13 256	13 220	–	1.014	–1.101
ILC or mixed ductal–lobular	0.085	0.479	0.176	0.860	–0.855	1.024	1.09	1.006	0.015
Previous contralateral cancer	0.816	0.655	1.245	0.213	–0.468	2.100	2.26	1.002	0.084
Previous ipsilateral cancer	1.305	0.510	2.560	0.010	0.306	2.305	3.69	1.006	0.144
T4 disease	2.228	0.337	6.606	< 0.001	1.567	2.889	9.28		
**Model 2 (*n* = 947), 5 variables, AUC 0.81**									
Constant	–6.303	1.076	–5.857	< 0.001	–8.412	–4.194			
Invasive ductal carcinoma	0.291	0.476	0.611	0.541	–0.643	1.225	1.34	1.009	0.068
Inflammatory cancer	2.690	0.617	4.361	< 0.001	1.481	3.899	14.73	1.015	0.173
Multifocality	0.189	0.410	0.461	0.645	–0.615	0.993	1.21	1.030	0.040
Symptomatic clinic attender	2.974	1.022	2.909	0.004	0.970	4.977	19.56	1.034	0.804
Symptoms of distant metastases	2.571	0.516	4.982	< 0.001	1.560	3.583	13.08	1.009	0.219
**Model 3 (*n* = 947), 6 variables, AUC 0.80**									
Constant	–4.334	0.319	–13.571	< 0.001	–4.960	–3.708			
Age ≤ 40 years	0.426	0.517	0.824	0.410	–0.587	1.440	1.53	1.062	0.055
Any abnormal nodes on ultrasonography	2.150	0.387	5.555	< 0.001	1.391	2.908	8.58	1.114	0.479
Central tumour	–0.047	0.498	–0.095	0.925	–1.024	0.930	0.95	1.047	–0.009
Symptoms of distant metastases	2.670	0.539	4.956	< 0.001	1.614	3.726	14.45	1.007	0.227
Synchronous bilateral cancers	0.023	0.771	0.030	0.976	–1.488	1.534	1.02	1.001	0.003
T3 disease	0.143	0.619	0.231	0.817	–1.070	1.356	1.15	1.061	0.017
**Model 4 (*n* = 947), 6 variables, AUC 0.82**									
Constant	–4.433	0.324	–13.693	< 0.001	–5.068	–3.799			
Grade 3	0.509	0.398	1.281	0.200	–0.270	1.289	1.66	1.311	0.128
ER-negative, HER2-positive	1.120	0.520	2.152	0.031	0.100	2.140	3.06	1.117	0.133
Lymphovascular invasion on initial core biopsy	–18.280	5235	–0.003	0.997	–10 279	10 243	–	1.020	–0.865
T4 disease	1.794	0.382	4.698	< 0.001	1.046	2.543	6.02	1.043	0.268
Triple-receptor negative	0.130	0.532	0.245	0.807	–0.913	1.173	1.14	1.192	0.022
Tumour ≥ 3 cm	1.416	0.380	3.730	< 0.001	0.672	2.161	4.12	1.105	0.335

VIF, variance inflation factor; AUC, area under receiver operating characteristic (ROC) curve; ILC, invasive lobular carcinoma; ER, oestrogen receptor; HER2, human epidermal growth factor receptor 2.

**Table 6:** Logistic regression coefficient estimates for models describing independent relationships between four variables and distant metastatic disease at presentation

	Coefficient	Standard error	*z*-statistic	*P*	Lower 95%	Upper 95%	Odds ratio	VIF	Standard coefficient
Constant	–4.335	0.287	–15.125	< 0.001	–4.897	–3.773			
ER-negative, HER2-positive	1.261	0.514	2.452	0.014	0.253	2.270	3.53	1.029	0.150
Symptoms of possible metastases	2.794	0.556	5.024	< 0.001	1.704	3.884	16.35	1.009	0.238
Tumour ≥ 3 cm and N1+	1.575	0.400	3.939	< 0.001	0.791	2.359	4.83	1.110	0.280
T4 disease	1.835	0.423	4.334	< 0.001	1.005	2.665	6.27	1.080	0.274

The analysis included 947 patients; area under receiver operating characteristic (ROC) curve 0.85. VIF, variance inflation factor; ER, oestrogen receptor; HER2, human epidermal growth factor receptor 2.

Sample sizes were small for lymphovascular invasion on core biopsy (7), inflammatory cancer (13), symptoms of possible metastatic disease (23), previous contralateral (36) or ipsilateral cancer (41), T3 disease (44), and ER-negative, HER2-positive disease (46).

The four variables with the greatest predictive value for distant metastatic disease were combined in the regression analysis presented in *Table 6*. Analysis of patients with tumour size >3cm and abnormal axillary nodes as a combined variable showed a greater positive predictive value for the presence of metastatic disease than each of these variables alone (PPV 18.8 per cent, OR 4.83, *P* <0.001, 115 patients).

### Data modelling

Thirty of the 47 patients with metastatic disease found in this study met widely used criteria for imaging, leaving 17 cases (36 per cent) potentially missed. Metastatic disease was identified in these 17 patients as a result of local evidence-based practice to perform CT staging for all patients with abnormal axillary nodes (accounting for 14 of 17 patients) and multidisciplinary team decisions to stage when considering neoadjuvant chemotherapy (accounting for the remaining 3).

Using data on all 1025 patients, models of the projected outcomes of different staging strategies were constructed (*Table 7*)*.* Modelling suggested that the addition of tumour size at least 3 cm with abnormal axillary nodes to existing staging criteria would lead to detection of a further nine patients (further 30 per cent) with distant metastatic disease. The models also suggest that this additional criterion would increase the positive CT rate to 17.1 per cent. With the subsequent addition of all patients referred for neoadjuvant chemotherapy, the model predicted that a further three patients with metastatic disease would be detected.

**Table 7:** Modelled data (1025 patients)

	Projected no. of pretreatment CT examinations	Metastatic disease detection rate of these CT examina tions (%)	Missed metastatic cases before treatment	Projected no. of postoperative CT examinations if performed for unexpected pN2+ disease	Metastatic disease detection rate of postoperative CT examinations
**Model 1: London criteria** T4 Recurrence Symptoms of possible metastases	151	19.9	17	86	7
**Model 2** T4 Recurrence Symptoms of possible metastases Tumour ≥ 3 cm and N1+	228	17.1	8	56	5
**Model 3** T4 Recurrence Symptoms of possible metastases Tumour ≥ 3 cm and N1+ Before NAC	260	16.2	5	53	6

NAC, neoaduvant chemotherapy.

## Discussion

Optimization of the criteria for CT staging is important in supporting identification of distant metastases, while minimizing potential harms including false-positives, unnecessary radiation dose, patient anxiety, and treatment delays.

Data from the present study indicate, like others, an increased risk of metastatic disease detection at presentation in those with symptoms suggestive of metastases, ipsilateral recurrent disease and T4 disease, but also a statistically significant increased risk in a selected group with tumour size at least 3 cm and concurrent abnormal axillary nodes. Another group^19^ has formed similar conclusions. The likelihood of CT detection of distant metastases in this additional patient group exceeded the rate of CT incidental findings/false-positives in the present study (14.6 per cent) and exceeds the threshold at which a majority of patients found it acceptable to undergo CT reported in a recent study^32^. Modelling suggests that its inclusion would reduce missed diagnoses of metastatic disease from an estimated 36 per cent to 17 per cent.

Analysis of patients based on their selection for neoadjuvant chemotherapy is confounded by the intrinsic links between the decision regarding such therapy, and features such as tumour size, nodal involvement, and receptor status. A significant proportion of these patients have metastatic disease at presentation and consideration of CT staging in this group seems reasonable. However, patient selection for neoadjuvant chemotherapy is evolving, so ongoing reassessment of this practice will be required.

Interestingly, T3 disease was not a statistically significant additional predictor of distant metastases in this study, although it is important to state that the sample size was small, reflecting the mix of screening and symptomatic patients in the cohort. In the present study, the risk of distant disease peaked at tumour size 3 cm or greater, with risk reducing again at tumour sizes of greater than 4 or 5 cm.

Triple-negative disease (ER-, progesterone receptor-, and HER2-negative) is typically of higher grade and more aggressive than tumours with other receptor profiles. Surprisingly, the present study did not demonstrate a significantly increased risk of detecting distant disease at presentation in this group. However, triple-negative disease was over-represented in the small cohort with a normal staging CT result at presentation who developed visible metastatic disease during follow-up, the incidence of distant metastases in this subgroup later increasing to 22 per cent. This may reflect established survival curves for triple-negative disease, representing the cohort of patients who respond poorly to treatment and show progressive disease.

Abnormal appearance of the axillary nodes on initial ultrasound imaging carried an increased risk of detectable metastatic disease, especially for tumour sizes of 3 cm or greater. The present study is particularly informative regarding the predictive value of nodal abnormalities as local evidence-based policy advocated staging where there was imaging, histological or cytological evidence of nodal involvement. Although this creates the potential for bias, as nodal abnormality may have been over-represented in the staged cohort, the average duration of follow-up serves to minimize bias by identifying patients who may have been under-represented by the staging policy.

Potential limitations of this study included a relatively small sample of patients with metastatic disease (47 patients initially, rising to 97 at follow-up), as the prevalence of metastatic disease is low and the study cohort reliably reflected this. There is also likely to have been selection bias, as already discussed. It would not be ethical to perform CT for every patient presenting with breast cancer and so a degree of bias in referral for staging is inevitable. An assumption must therefore also be made that any patient not referred for CT staging at presentation does not have detectable metastatic disease. This is unlikely to be true for all patients, bearing in mind those who are proven to have metastatic disease at follow-up.

On the basis of the present results, currently accepted criteria for CT imaging to detect distant metastatic disease in patients with symptoms of possible metastatic disease, ipsilateral recurrent breast cancer and T4 disease continue to be appropriate. In addition, consideration should be given to CT in patients with a primary tumour size of at least 3 cm and a concurrent nodal abnormality on axillary ultrasonography.

CT staging should also be considered before commencing neoadjuvant chemotherapy, if not meeting the above criteria, although this aspect of staging practice requires regular re-evaluation given the gradual shift towards preoperative management with neoadjuvant chemotherapy.

## Supplementary Material

zraa006_Supplementary_Data

## Notes

Presented to the British Society of Breast Radiology Annual Scientific Meetings in November 2017 and November 2019 respectively
