# Supplementary material for: Expanded criteria for pretreatment staging CT in breast cancer
Source: BJS Open. 2021 Mar 14;5(2):zraa006. doi: 10.1093/bjsopen/zraa006 (PMC7955978; doi:10.1093/bjsopen/zraa006)
Supplement: zraa006_Supplementary_Data [file zraa006_supplementary_data.zip › Suppl_info_1_Roszkowski_et_al.docx]

**CT STAGING PROJECT – DATA COLLECTION PROFORMA**

Collect **only invasive carcinoma** (not DCIS or Pagets), who stay at UHS for assessment and treatment (not PP or gone to DGH). **Keep a separate record of patients excluded** from data collection and why.

**PROFORMA NUMBER:**

**DEMOGRAPHICS:**

| Month & year of presentation |  |
| --- | --- |
| Hospital number: | RHM |
| JSO number (if screening): | JSO |

| DOB & age: |  |
| --- | --- |
| Gender: |  |
| Screening or symptomatic? |  |

| Diagnosed incidentally on CT? Y or N | |  |
| --- | --- | --- |
| CT’d for **staging** purposes? When?  (Pre-op, pre-NAC or post-op? Within 3 months?) | |  |
| Indication for staging? Symptoms? | |  |
| Findings on staging CT – Completely normal? Malig nodes? Distant mets? Where are mets? Indeterminate findings requiring further invx? What invx and what was outcome? Benign or malignant? |  | |
| New mets or recurrence found on any subsequent imaging? (Y or N? When? What?) |  | |
| How long is the follow-up period? (date of presentation – now) | months | |

**CLINICAL & IMAGING:**

| Family history? (none, high, mod or low risk) |  |
| --- | --- |
| Previous breast cancer? (side? when?) |  |
| Size of largest focus on MM or US | mm |
| Unifocal or multifocal? |  |
| Central (i.e. retroareolar) or peripheral or both? |  |
| Skin or chest wall involvement or both? Or none? |  |
| Inflammatory cancer? Y or N |  |
| Axillary nodes on US? (Normal, equivocal, 1 abnormal, >1 abnormal, or nodal mass >25mm?) |  |
| Node sampling? (none, FNA or core biopsy?) |  |

| Post op size type & grade |  | | |
| --- | --- | --- | --- |
| No. nodes resected |  | No. nodes positive (micro, macro or ECS?) |  |

**PRE-OP BIOPSY PATH:**

**POST-OP PATH:**

| Type of invasive cancer? |  |
| --- | --- |
| Grade |  |
| ER, HER2, HER2 ISH, PR statuses |  |
| Lymphovasc invasion? |  |
| Node FNA or CB result (if applicable) |  |

**MANAGEMENT**

| Management pathway? (primary operative? primary hormones? NAC? operation later in pathway? palliative from outset? |  |
| --- | --- |
