# Supplementary material for: Expanded criteria for pretreatment staging CT in breast cancer
Source: BJS Open. 2021 Mar 14;5(2):zraa006. doi: 10.1093/bjsopen/zraa006 (PMC7955978; doi:10.1093/bjsopen/zraa006)
Supplement: zraa006_Supplementary_Data [file zraa006_supplementary_data.zip › Suppl_info_2_Roszkowski_et_al.docx]

**Supplementary file 2**

**Study Flow Diagram**

## Analysis

## Data Collection

Excluded **(n=352)**

♦  Incomplete data (treatment elsewhere) (n=351 )

♦  Incomplete data reason unknown (n=1)

Initial binomial logistic regression analyses **(n=988)**

Excluded **(n=37)**

♦ not imaged (n=1)

♦ occult tumour (n=5)

♦ numerical tumour size not recorded (n=13)

♦ no axillary ultrasound (n=7)

♦ no breast core biopsy (n=2)

♦ lost to follow-up abroad (n=1)

♦ patient has another concurrent metastatic cancer during our study period (n=10)

Excluded **(n=41)**
♦ Previous ipsilateral breast cancer (as this may be recurrent rather than new disease)

Final binomial logistic regression analyses **(n=947)**

Initial analysis **(n=1025)**

Demographic analysis and simple calculations of incidence, PPV, etc

All patients presenting over a 3 year period **(n=1377 )**
