# Supplementary material for: Expanded criteria for pretreatment staging CT in breast cancer
Source: BJS Open. 2021 Mar 14;5(2):zraa006. doi: 10.1093/bjsopen/zraa006 (PMC7955978; doi:10.1093/bjsopen/zraa006)
Supplement: Supplementary file 3 [file zraa006_supplementary_data.zip › TAbles.odt]

| Table 1: Summarised current European guidelines on systemic staging for breast cancer |
| --- |
| Source | Summarised guidance on which patients should be staged |
| NICE CG813 | Assess for the presence and extent of metastases in advanced breast cancer (stage IV) using appropriate modalities |
| NICE QS124 | People with early invasive breast cancer do not undergo staging investigations for distant metastatic disease in the absence of symptoms. |
| Royal College of Radiologists (RCR)20 | Indications for staging: T3 and T4 primary cancers >4 abnormal nodes at axillary ultrasound or >4 macrometastatic nodes at axillary surgery If symptoms raise the suspicion of metastatic disease At present, there is no evidence base for carrying out staging prior to neoadjuvant chemotherapy in <T2 tumours with <N1 disease |
| London Cancer Surgical Guidelines21 | Indications for staging using CT scan and isotope bone scan: Symptoms suggestive of metastases Recurrent disease Significant nodal involvement (e.g. ≥4 nodes) As part of an approved clinical trial Inflammatory breast cancer Locally advanced disease Large primary tumours (e.g. >5cm) |
| ESMO5 | Staging can be considered for patients with: Clinically positive axillary nodes Large tumours (e.g. ≥5cm) Aggressive biology Clinical signs, symptoms or laboratory values suggesting presence of metastases |
| ABS19 | Consider staging for: Recurrent breast cancer Symptoms suggesting possible metastases Partaking in a clinical trial Recommended for neo-adjuvant therapy |
| NCCN32 | Consider staging with body CT, bone scan and (optional) PET-CT for: Signs or symptoms of possible metastases Stage IV disease Inflammatory breast cancer Work-up prior to pre-operative systemic therapy >4 positive axillary nodes at surgery Routine systemic staging is not indicated for early breast cancer in the absence of symptoms |

| Table 2: Patient features and disease characteristics of the 1025 patients in the dataset |
| --- |
| Features at presentation | n (% of whole cohort) | Percentage staged with CT pre-treatment or post-operatively |
| Personal or family histories |
| Previous ipsilateral breast cancer | 51 (5.0%) | 76.5% |
| Previous contralateral breast cancer | 38 (3.7%) | 55.3% |
| Low or moderate risk family history | 119 (11.6%) | 29.4% |
| High risk family history (including BRCA) | 12 (1.2%) | 75.0% |
| T stage |
| T1 | 482 (47.0%) | 19.1% |
| T2 | 389 (38.0%) | 36.5% |
| T3 | 45 (4.4%) | 51.1% |
| T4 | 86 (8.4%) | 65.1% |
| Specific imaging characteristics |
| Inflammatory cancer | 13 (1.3%) | 100% |
| Multifocal cancer | 185 (18.0%) | 50.3% |
| Abnormal or equivocal axillary nodes on US | 267 (26.0%) | 73.0% |
| Histology |
| Invasive ductal carcinoma of no special type | 777 (75.8%) | 34.4% |
| Invasive lobular carcinoma | 87 (8.5%) | 25.3% |
| Mixed or other disease | 161 (15.7%) | 21.1% |
| Histologic grade |
| Grade 1 | 158 (15.4%) | 17.7% |
| Grade 2 | 536 (52.3%) | 28.0% |
| Grade 3 | 295 (28.8%) | 49.2% |
| Specific molecular subtypes |
| Triple receptor negative | 104 (10.1%) | 55.8% |
| All HER2 positive disease | 114 (11.1%) | 37.7% |
| ER negative, HER2 positive | 48 (4.7%) | 52.1% |
| Other features |
| Symptoms of possible metastatic disease at presentation (e.g. bone pain) | 29 (2.8%) | 89.7% |

| Table 3: The incidence of metastatic disease for specific variables |
| --- |
| Variable | n | % initially staged | Incidence of detected distant metastases |
| At presentation | After 26.8 months follow-up |
| Inflammatory breast cancer | 13 | 100% | 46.2% | 61.5% |
| Symptoms of possible metastases | 23 | 100% | 34.8% | 65.2% |
| T4 disease | 75 | 74.7% | 21.3% | 32.0% |
| Tumour size ≥3cm and N1+ | 115 | 86.1% | 18.8% | 34.8% |
| HER2 +ve, ER -ve | 46 | 54.3% | 17.4% | 26.1% |
| T2+ N1+ | 154 | 85.1% | 16.9% | 32.5% |
| Before neo-adjuvant chemotherapy | 78 | 96.2% | 16.7% | 30.8% |
| Ipsilateral recurrence | 41 | 95.1% | 14.6% | 29.3% |
| N1+ on axillary US | 176 | 80.7% | 13.8% | 31.3% |
| Age ≤40 years | 54 | 66.7% | 11.1% | 29.6% |
| T3 disease | 44 | 52.3% | 9.1% | 18.2% |
| Previous contralateral cancer | 36 | 58.3% | 8.3% | 13.9% |
| Grade 3 | 295 | 49.2% | 8.0% | 19.7% |
| Multifocal disease | 172 | 54.1% | 6.4% | 16.9% |
| Triple receptor negative | 98 | 59.2% | 6.1% | 22.4% |
| T3 N0 disease | 19 | 15.8% | 5.9% | 15.8% |
| Lobular carcinoma | 85 | 25.9% | 5.9% | 11.8% |
| Family history of breast cancer | 126 | 34.9% | 0.8% | 8.7% |
| Lymphovascular invasion in core | 7 | 85.7% | 0% | 14.3% |

| Table 4: Tumour size and nodal appearances - positive predictive values (PPV) for distant metastatic disease (DMD) |
| --- |
| Variable | n | PPV for DMD |
| Tumour size (T stage disregarded) |
| Less than 2cm | 482 | 1.2% |
| 2cm or more | 483 | 7.2% |
| 3cm or more | 229 | 11.4% |
| 4cm or more | 115 | 11.3% |
| 5cm or more | 44 | 9.1% |
| Nodal appearances |
| Normal | 748 | 1.9% |
| 1 abnormal node only | 82 | 11.0% |
| >1 (multiple) abnormal nodes | 113 | 15.9% |
| Axillary nodal mass >25mm | 12 | 8.3% |

| # Table 5: Logistic regression coefficients estimates for four different models |
| --- |
| # Model 1: Six variables and their independent relationship with distant metastatic disease at presentation. Area under ROC curve = 0.71 (n=988). Statistically significant values highlighted. |
| # Variable | # Coefficient | # Standard error | # z-statistic | # p-value | # Lower 95% | # Upper 95% | # Odds ratio | # VIF | # Standard coefficient |
| # Constant | # -3.479 | # 0.267 | # -13.054 | # 0.000 | # -4.002 | # -2.957 |  |  |  |
| # Age 60 or over | # -0.252 | # 0.325 | # -0.777 | # 0.437 | # -0.889 | # 0.385 | # 0.777 | # 1.019 | # -0.069 |
| # High risk family history | # -18.216 | # 6,754 | # -0.003 | # 0.998 | # -13,256 | # 13,220 | # - | # 1.014 | # -1.101 |
| # ILC or mixed ductal-lobular | # 0.085 | # 0.479 | # 0.176 | # 0.860 | # -0.855 | # 1.024 | # 1.088 | # 1.006 | # 0.015 |
| # Previous contralateral cancer | # 0.816 | # 0.655 | # 1.245 | # 0.213 | # -0.468 | # 2.100 | # 2.262 | # 1.002 | # 0.084 |
| # Previous ipsilateral cancer | # 1.305 | # 0.510 | # 2.560 | # 0.010 | # 0.306 | # 2.305 | # 3.689 | # 1.006 | # 0.144 |
| # T4 disease | # 2.228 | # 0.337 | # 6.606 | # 0.000 | # 1.567 | # 2.889 | # 9.280 |  |  |
| # Model 2: Five variables and their independent relationship with distant metastatic disease at presentation. Area under ROC curve = 0.81 (n=947).  Statistically significant values highlighted. |
| # Variable | # Coefficient | # Standard error | # z-statistic | # p-value | # Lower 95% | # Upper 95% | # Odds ratio | # VIF | # Standard coefficient |
| # Constant | # -6.303 | # 1.076 | # -5.857 | # 0.000 | # -8.412 | # -4.194 |  |  |  |
| # Invasive ductal carcinoma | # 0.291 | # 0.476 | # 0.611 | # 0.541 | # -0.643 | # 1.225 | # 1.338 | # 1.009 | # 0.068 |
| # Inflammatory cancer | # 2.690 | # 0.617 | # 4.361 | # 0.000 | # 1.481 | # 3.899 | # 14.733 | # 1.015 | # 0.173 |
| # Multifocality | # 0.189 | # 0.410 | # 0.461 | # 0.645 | # -0.615 | # 0.993 | # 1.208 | # 1.030 | # 0.040 |
| # Symptomatic clinic attender | # 2.974 | # 1.022 | # 2.909 | # 0.004 | # 0.970 | # 4.977 | # 19.562 | # 1.034 | # 0.804 |
| # Symptoms of distant metastases | # 2.571 | # 0.516 | # 4.982 | # 0.000 | # 1.560 | # 3.583 | # 13.079 | # 1.009 | # 0.219 |

| # Model 3: Six variables and their independent relationship with distant metastatic disease at presentation. Area under ROC curve = 0.80 (n=947).  Statistically significant values highlighted. |
| --- |
| # Variable | # Coefficient | # Standard error | # z-statistic | # p-value | # Lower 95% | # Upper 95% | # Odds ratio | # VIF | # Standard coefficient |
| # Constant | # -4.334 | # 0.319 | # -13.571 | # 0.000 | # -4.960 | # -3.708 |  |  |  |
| # Age 40 or less | # 0.426 | # 0.517 | # 0.824 | # 0.410 | # -0.587 | # 1.440 | # 1.531 | # 1.062 | # 0.055 |
| # Any abnormal nodes on US | # 2.150 | # 0.387 | # 5.555 | # 0.000 | # 1.391 | # 2.908 | # 8.583 | # 1.114 | # 0.479 |
| # Central tumour | # -0.047 | # 0.498 | # -0.095 | # 0.925 | # -1.024 | # 0.930 | # 0.954 | # 1.047 | # -0.009 |
| # Symptoms of distant mets | # 2.670 | # 0.539 | # 4.956 | # 0.000 | # 1.614 | # 3.726 | # 14.445 | # 1.007 | # 0.227 |
| # Synchronous bilateral cancers | # 0.023 | # 0.771 | # 0.030 | # 0.976 | # -1.488 | # 1.534 | # 1.023 | # 1.001 | # 0.003 |
| # T3 disease | # 0.143 | # 0.619 | # 0.231 | # 0.817 | # -1.070 | # 1.356 | # 1.154 | # 1.061 | # 0.017 |
| # Model 4: Six variables and their independent relationship with distant metastatic disease at presentation. Area under ROC curve = 0.82 (n=947).  Statistically significant values highlighted. |
| # Variable | # Coefficient | # Standard error | # z-statistic | # p-value | # Lower 95% | # Upper 95% | # Odds ratio | # VIF | # Standard coefficient |
| # Constant | # -4.433 | # 0.324 | # -13.693 | # 0.000 | # -5.068 | # -3.799 |  |  |  |
| # Grade 3 | # 0.509 | # 0.398 | # 1.281 | # 0.200 | # -0.270 | # 1.289 | # 1.664 | # 1.311 | # 0.128 |
| # ER negative HER2 positive | # 1.120 | # 0.520 | # 2.152 | # 0.031 | # 0.100 | # 2.140 | # 3.064 | # 1.117 | # 0.133 |
| # Lymphovascular invasion on initial core biopsy | # -18.280 | # 5,235 | # -0.003 | # 0.997 | # -10,279 | # 10,243 | # - | # 1.020 | # -0.865 |
| # T4 disease | # 1.794 | # 0.382 | # 4.698 | # 0.000 | # 1.046 | # 2.543 | # 6.016 | # 1.043 | # 0.268 |
| # Triple receptor negative | # 0.130 | # 0.532 | # 0.245 | # 0.807 | # -0.913 | # 1.173 | # 1.139 | # 1.192 | # 0.022 |
| # Tumour 3cm or more | # 1.416 | # 0.380 | # 3.730 | # 0.000 | # 0.672 | # 2.161 | # 4.122 | # 1.105 | # 0.335 |

| # Table 6: Logistic regression coefficients estimates. Four variables and their independent relationship with distant metastatic disease at presentation. Area under ROC curve = 0.85 (n=947) |
| --- |
| # Variable | # Coefficient | # Standard error | # z-statistic | # p-value | # Lower 95% | # Upper 95% | # Odds ratio | # VIF | # Standard coefficient |
| # Constant | # -4.335 | # 0.287 | # -15.125 | # 0.000 | # -4.897 | # -3.773 |  |  |  |
| # ER-ve HER2+ve | # 1.261 | # 0.514 | # 2.452 | # 0.014 | # 0.253 | # 2.270 | # 3.530 | # 1.029 | # 0.150 |
| # Symptoms of possible mets | # 2.794 | # 0.556 | # 5.024 | # 0.000 | # 1.704 | # 3.884 | # 16.346 | # 1.009 | # 0.238 |
| # Tumour ≥3cm and N1+ | # 1.575 | # 0.400 | # 3.939 | # 0.000 | # 0.791 | # 2.359 | # 4.831 | # 1.110 | # 0.280 |
| # T4 disease | # 1.835 | # 0.423 | # 4.334 | # 0.000 | # 1.005 | # 2.665 | # 6.267 | # 1.080 | # 0.274 |

| Table 7: Modelled data (n=1025) |
| --- |
| Staging model | Projected number of pre-treatment CTs | Metastatic disease detection rate of these CTs | Missed metastatic cases pre-treatment | Projected number of post-operative CTs if performed for unexpected pN2+ | Metastatic disease detection rate of post-operative CTs |
| Model 1: London Criteria T4 Recurrence Symptoms of poss mets | 151 | 19.9% | 17 | 86 | 7.0% |
| Model 2: T4 Recurrence Symptoms of poss mets Tumour ≥3cm & N1+ | 228 | 17.1% | 8 | 56 | 5.4% |
| Model 3: T4 Recurrence Symptoms of poss mets Tumour ≥3cm & N1+ Before NAC | 260 | 16.2% | 5 | 53 | 5.7% |
